# A TaqMan-based multiplex real-time PCR assay for the rapid detection of tigecycline resistance genes from bacteria, faeces and environmental samples

**DOI:** 10.1186/s12866-020-01813-8

**Published:** 2020-06-22

**Authors:** Yiming Li, Zhangqi Shen, Shuangyang Ding, Shaolin Wang

**Affiliations:** 1 Beijing Advanced Innovation Center for Food Nutrition and Human Health, College of Veterinary Medicine, China Agricultural University, Beijing, China; 2 Beijing Key Laboratory of Detection Technology for Animal Derived Food Safety and Beijing Laboratory for Food Quality and Safety, Beijing, China

**Keywords:** Tigecycline resistance, TaqMan, Real-time PCR

## Abstract

**Background:**

Tigecycline is a last-resort antibiotic used to treat severe infections caused by extensively drug-resistant bacteria. Recently, novel tigecycline resistance genes *tet*(X3) and *tet*(X4) have been reported, which pose a great challenge to human health and food security. The current study aimed to establish a TaqMan-based real-time PCR assay for the rapid detection of the tigecycline-resistant genes *tet*(X3) and *tet*(X4).

**Results:**

No false-positive result was found, and the results of the TaqMan-based real-time PCR assay showed 100% concordance with the results of the sequencing analyses. This proposed method can detect the two genes at the level of 1 × 10^2^ copies/μL, and the whole process is completed within an hour, allowing rapid screening of *tet*(X3) and *tet*(X4) genes in cultured bacteria, faeces, and soil samples*.*

**Conclusion:**

Taken together, the TaqMan-based real-time PCR method established in this study is rapid, sensitive, specific, and is capable of detecting the two genes not only in bacteria, but also in environmental samples.

## Background

With the prevalence of antimicrobial resistance (AMR), only a few antibiotics are available to treat severe infections caused by extensively drug-resistant (XDR) bacteria, which poses a great challenge to human health and food security. Tigecycline and colistin are last-resort drugs to treat infections caused by carbapenem-resistance *Enterobacteriaceae* [1]. The World Health Organization (WHO) classified the two antibiotics as critically important antimicrobials, and their usage should be severely restricted (http://www.who.int/foodsafety/publications/antimicrobials-fifth/en/) [2]. Since the recent reports of colistin resistance genes (*mcr*), the clinical application of colistin has become more limited [2, 3], turning tigecycline into the ultimate treatment option.

In May 2019, two tigecycline resistance genes, *tet*(X3) and *tet*(X4), were discovered, which can inactivate the entire family of tetracycline antibiotics, including tigecycline and the newly US FDA-approved drugs eravacycline and omadacycline [4]. *Tet*(X3) and *tet*(X4) are the first plasmid-borne *tet*(X) genes, encoding proteins with 386 amino acids and 385 amino acids, respectively, and showing 85.1 and 94.3% identity, respectively, with the original *tet*(X) from *Bacterioides fragilis* [4, 5].

To date, both genes have been identified in humans, animals, meat, and environmental samples [4, 6–8]. In three representative provinces of China, *tet*(X3) and *tet*(X4) genes have been identified in animals and meat for consumption at a high proportion of 43.3% [4], indicating the wide transmission of tigecycline resistance. Recent studies showed that the presence of *tet*(X3) and *tet*(X4) genes can significantly increase the resistance to tigecycline, and the construction of a *tet*(X4)-containing bacterial strain, namely, *Escherichia coli* JM109 + pBAD24-tet(X4), increases the MIC value of tigecycline by 64-fold compared with the original host strain [8].

Therefore, it is necessary to establish an efficient method for simultaneous screening the tigecycline-resistant genes *tet*(X3) and *tet*(X4) in different samples. Nowadays, real-time PCR assays are widely used in laboratories domestically and overseas. Compared to conventional PCR, real-time PCR has superior sensitivity, reproducibility, precision, and high throughput capability [9]. In this study, we designed a rapid, sensitive TaqMan-based multiplex real-time PCR assay for the specific detection of the tigecycline resistance genes *tet*(X3) and *tet*(X4), and further evaluated using cultured bacteria, faeces and soil samples.

## Results

### Primers and probes

**Fig. 1:**
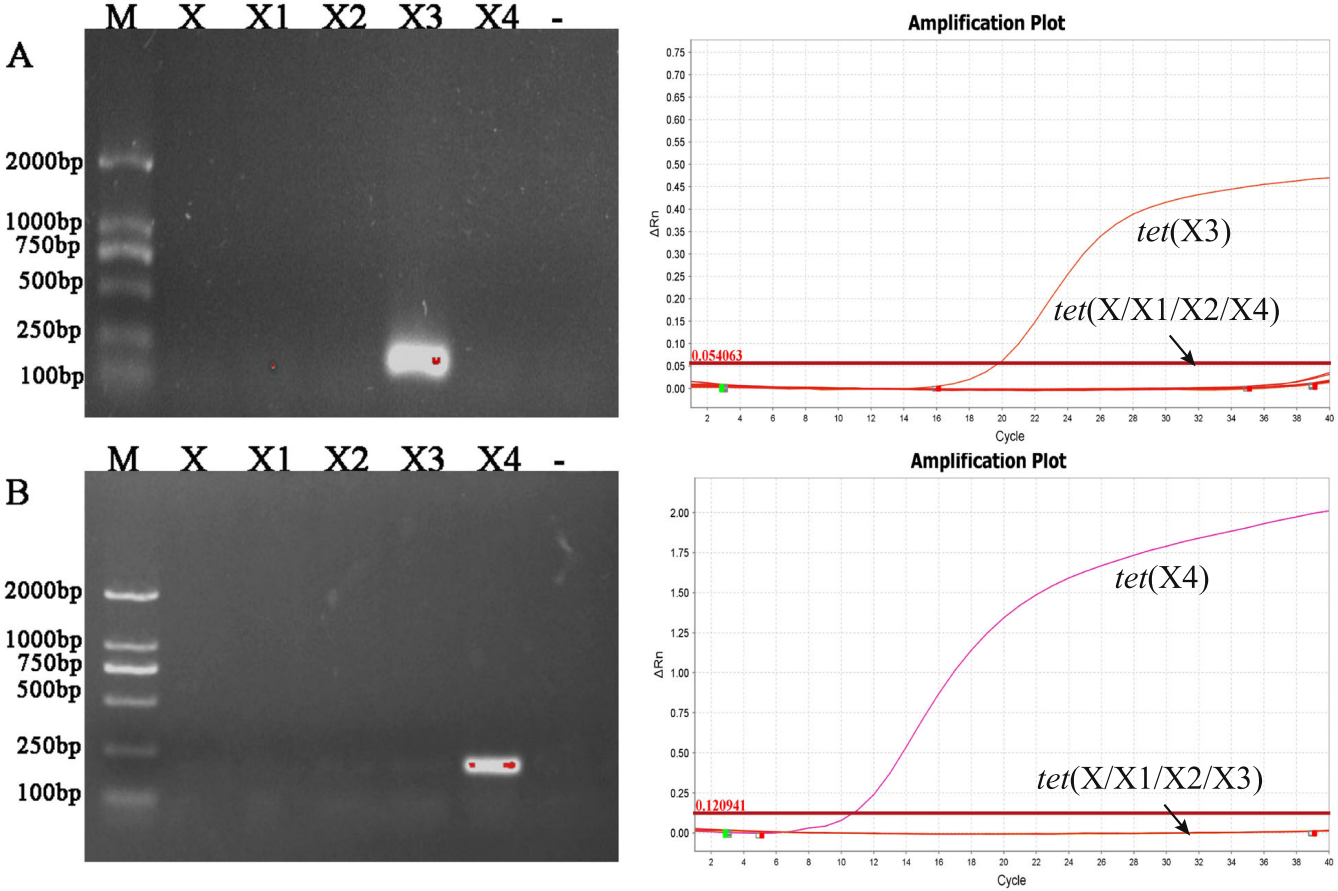
Conventional PCR amplification and real-time PCR amplification curve for *tet*(X3) *and te*t(X4) genes. **a–b** were the electrophoresis gel (left) and amplification curve (right) of *tet*(X3) *and tet*(X4). M: Marker. -: negative control. X3 was *tet*(X3) positive strain. X4 was *tet*(X4) positive strain. X, X1, X2 were negative strains

The results of the NCBI Primer BLAST module indicated that no genes other than *tet*(X3) and *tet*(X4) genes matched the primer sequences designed in this study. Similarly, the results of conventional and real-time PCR also indicated the high specificity of primers and probes (Fig. 1).

### Real-time PCR and standard curve analysis

Standard curves were obtained using 10-fold serial dilutions of plasmids pTET(X3) and pTET(X4), containing the *tet*(X3) and *tet(*X4) genes to determine the detection limit of the proposed method. The detection range of copies was 1.49 × 10^2^–1.49 × 10^10^ copies/μL for *tet*(X3) and 1.23 × 10^2^–1.23 × 10^10^ copies/μL for *tet*(X4*)*, and cycle threshold (CT) ranges were 37.435–9.663 for *tet*(X3) and 36.894–9.273 for *tet*(X4).

**Fig. 2:**
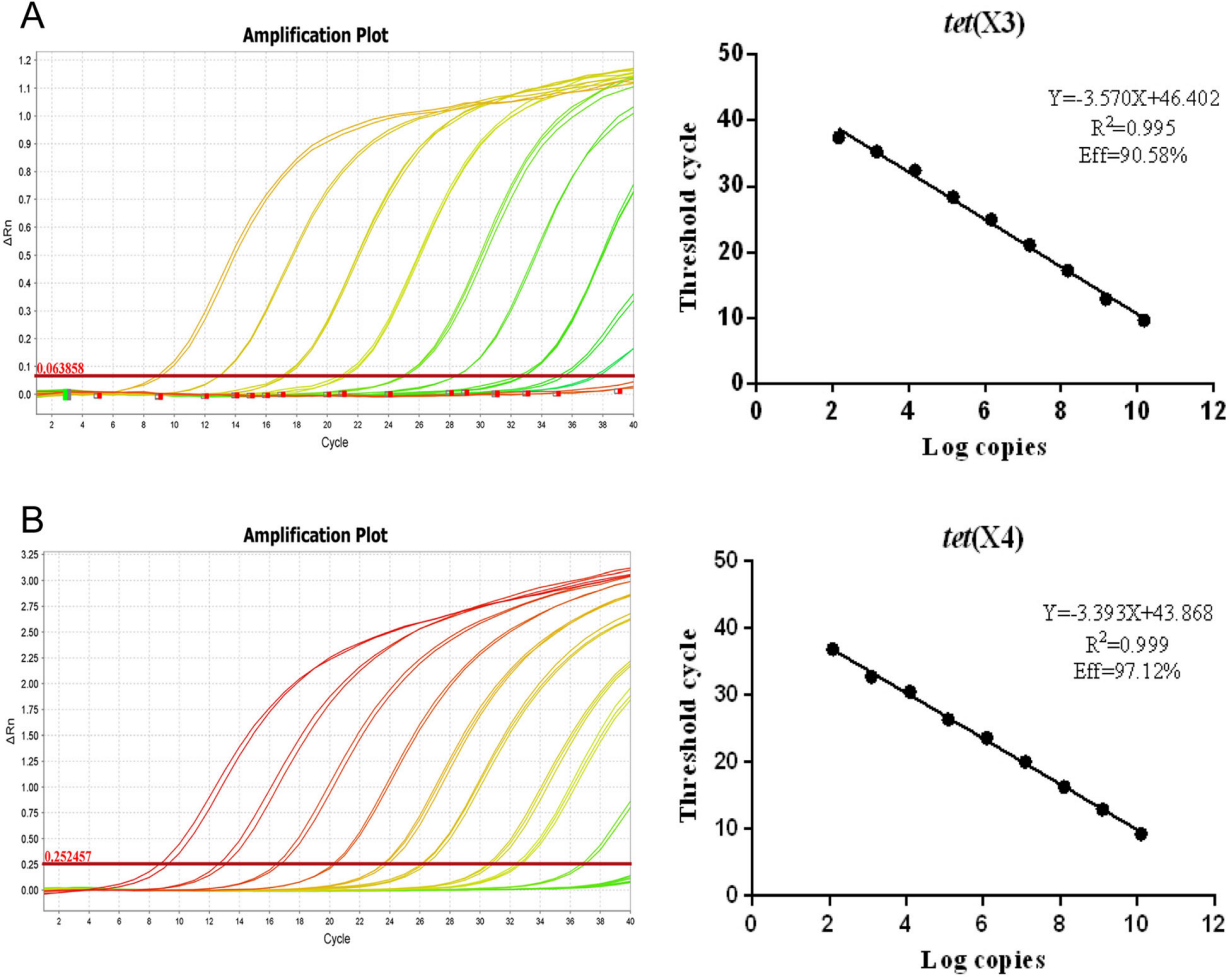
Real-time PCR amplification curves and standard curves. **a–b** Show real-time PCR amplification curves and standard curves *tet*(X3) and *tet*(X4)

Linear standard curves for real-time PCR are shown in Fig. 2. The amplification efficiencies were calculated using the formula *E* = 10^(− 1/slope)^ − 1 [10]. R^2^ values were 0.995 and 0.999, respectively, and efficiency was 90.58 and 97.12% for the *tet*(X3*)* and *tet*(X4) genes, respectively. The sensitivity of analysis was linear within the dynamic range of 9 dilutions. These results reveal that these two real-time PCR tests are accurate for quantitative detection of *tet*(X3) and *tet*(X4) genes.

### Specificity and sensitivity evaluation

To confirm the specificity of the assay, two *E. coli* DH5α strains carrying *tet*(X3) or *tet*(X4) were used as the positive control, while *E. coli* ATCC25922 and three *E. coli* DH5α strains containing *tet*(X), *tet*(X1) or *tet*(X2), respectively, were used as the negative control. Each sample was tested three times independently (*n* = 3). The results of TaqMan-based real-time PCR were 100% concordance with the results of conventional PCR (Table 1**&** Fig. 1), which proved that the two primer sets and probes were highly specific for their target gene.

**Table 1:** Detection of *tet*(X3) and *tet*(X4) genes in isolates

Isolate	Origin	Species	Gene	Gene Location	Real-time PCR for *tet(X)*		
					*tet*(X3)	*tet*(X4)	Ct ± SD
ATCC25922	–	*E. coli*	–	–	–	–	Undetermined
DH5α-*tet*(X)	–	*E. coli*	*tet*(X)	Plasmid	–	–	Undetermined
DH5α-*tet*(X1)	–	*E. coli*	*tet*(X1)	Plasmid	–	–	Undetermined
DH5α-*tet*(X2)	–	*E. coli*	*tet*(X2)	Plasmid	–	–	Undetermined
DH5α-*tet(X3)*	–	*E. coli*	*tet*(X3)	Plasmid	+	–	9.66 ± 0.00
DH5α-*tet(X4)*	–	*E. coli*	*tet*(X4)	Plasmid	–	+	9.27 ± 0.00
CB13	Chicken	*E. coli*	*tet*(X3)	Plasmid	+	–	19.05 ± 0.05
CB14	Chicken	*E. coli*	*tet*(X3)	Plasmid	+	–	21.06 ± 0.08
CB15	Chicken	*E. coli*	*tet*(X3)	Plasmid	+	–	19.24 ± 0.10
CB42	Chicken	*E. coli*	*tet*(X3)	Plasmid	+	–	19.24 ± 0.16
DZ47	Chicken	*E. coli*	*tet*(X4)	Plasmid	–	+	21.90 ± 0.12
AZ28	Chicken	*E. coli*	*tet*(X4)	Plasmid	–	+	14.79 ± 0.01
DZ4R	Chicken	*A. baumannii*	*tet*(X4)	Plasmid	–	+	13.42 ± 0.07
NM4	Chicken	*A. baumannii*	*tet*(X4)	Plasmid	–	+	14.23 ± 0.13
DZ27	Chicken	*E. coli*	*tet*(X4)	Plasmid	–	+	17.76 ± 0.13
DZ24	Chicken	*E. coli*	*tet*(X4)	Plasmid	–	+	14.35 ± 0.10
DZ65	Chicken	*A. baumannii*	*tet*(X4)	Plasmid	–	+	21.05 ± 0.05
DZ24	Chicken	*E. coli*	*tet*(X4)	Plasmid	–	+	18.33 ± 0.09

**Fig. 3:**
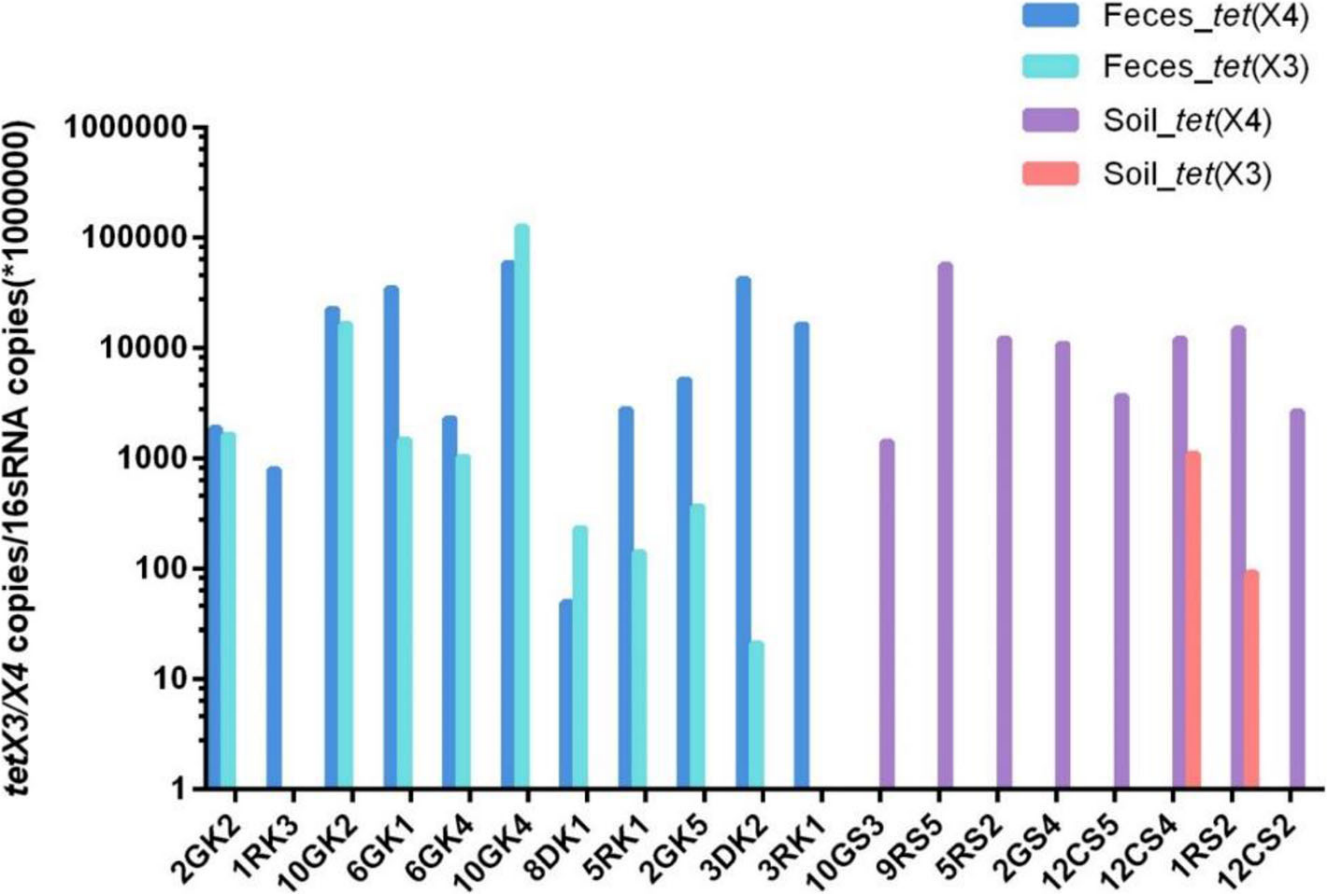
Detection of *tet*(X3) and *tet*(X4) genes in faeces and soil samples. The relative abundance of *tet*(X3) and *tet*(X4) genes (copies /per 1,000,000 copies of 16S rRNA) in the soil and faeces samples

To further validate the method, genetic DNA extracted from bacteria, faeces and soil samples (three independent technical replicates) was selected to conduct the real-time PCR assay for screening. In this study, some *E. coli* and *A. baumannii* isolates of animal origin were selected for verification, including 4 *tet*(X3) positive strains and 8 *tet*(X4) positive strains (Table 1). The CT ranges were 19.05–21.06, 13.42–21.05 for *tet*(X3), *tet*(X4) genes (Table 1). The results of real-time PCR and previous sequencing analyses were completely consistent, showing high specificity of the method. Moreover, a total of 24 faeces and soil samples from chickens, pigs, and cattle were collected for further evaluation. We were able to detect the two genes in metagenomes extracted from 19 faeces and soil samples, and the relative abundance was normalized using 16S rRNA (gene copies/10^6^ copies of the 16S rRNA gene) [11, 12]. The real-time PCR assay showed that the normalized copies range from 10^1^ to 10^5^ for genes *tet*(X3) and *tet*(X4) (Fig. 3).

## Discussion

Since the first discovery of the *tet*(X3) and *tet*(X4) genes, these two plasmid-mediated tigecycline resistance genes have been widely reported, suggesting that they are spreading at an alarming rate. It is noteworthy that the *tet*(X3) and *tet*(X4) genes have been identified not only in humans and animals, but also in the environment [4, 13, 14]. There is a pressing need to establish a fast screening assay for tigecycline resistance genes. So far, there are three reports of fast screening of *tet*(X3) and *tet*(X4), multiplex PCR methods, tetracycline inactivation method, and SYBR Green based real-time PCR, all three methods have approved the effectiveness of methods [15–17]. Compared to traditional detection methods, like conventional PCR and phenotypic method, real-time PCR assays are more sensitive, specific, time-efficient, and labour-saving [18]. Besides, real-time PCR methods can detect genes in different type of samples other than bacteria. Lately, Fu et al. reported a SYBR Green-based real-time PCR assay for rapid detection of *tet*(X) variants, with a detection limit range from 10^2^ to 10^5^, 1–10^3^ per 10^6^ copies of 16S rRNA for *tet*(X3) and *tet*(X4), respectively. To date, the method based on real-time PCR using TaqMan probe has not been previously proposed.

In this study, we developed a TaqMan-based multiplex real-time PCR assay for the detection of the *tet*(X3) and *tet*(X4) genes. Both *tet*(X3) and *tet*(X4) genes have been successfully identified not only in bacteria isolates, but also directly from faeces and soil samples, with a minimum of 1 copy per 10^5^ copies of 16S rRNA and a maximum of 10^4^ copies per 10^5^ copies of 16S rRNA. Besides, we used constructed *E. coli* DH5α-*tet*(X) strains as positive control and bacteria isolates of animal origin to evaluate the specificity of the method. The *E. coli* ATCC25922 was used as negative control. In our method, only specific amplicons can be bonded by TaqMan probes, which is different from the SYBR-Green dye. The SYBR-Green can bind any double strand DNA fragments in the PCR reaction without any specificity, so melting curve analysis will be necessary to identify the single peak for the PCR reaction. According to the Fig. 1, our method has high specificity, whereas other *tet*(X) genes couldn’t be amplified. A limitation of the proposed method is that these genes cannot be detected simultaneously in a single reaction. However, there are no reports of the co-existence of these two genes. Because *tet*(X3) and *tet*(X4) genes are usually accompanied with MDR [9], different combinations of such detection methods are flexible and convenient.

Although tigecycline usage has never been approved in animal husbandry, tetracyclines have been widely used in China and many other countries. The total consumption of tetracyclines reached 13,666 tons in 2018 in China, which may provide ongoing selective pressure for the production of tigecycline resistance genes. Many studies have shown that *tet*(X4) can be captured by a range of mobile elements circulating among bacterial strains [4, 6–8], importantly, with the international trade of food-producing animals and their derivatives, a novel antibacterial mechanism may appear. It is of great importance to monitor and eradicate these genes, especially in countries with high tetracycline consumption.

## Conclusion

Overall, we developed a TaqMan-based multiplex real-time PCR method in this study for the rapid detection of tigecycline resistance genes, *tet*(X3) and *tet*(X4). This assay can be widely applied to all laboratories equipped with a qPCR machine, and the whole process could be completed within an hour. It is highly sensitive and specific, and can detect and quantify *tet*(X3) and *tet*(X4) genes accurately in cultured bacteria isolates, faeces and environmental samples.

## Methods

### Bacteria strains and environmental samples

All the *E. coli* and *A. baumannii* strains from animal origin used in this study (Table 1) were collected from three poultry farm in Shandong Province, and were identified by conventional PCR and MALDI-TOF before. Five *tet*(X) variant genes were cloned into the PMD-19 T vector (Takara Bio, Kusatsu, Japan), and then transferred into the DH5α cell, including DH5α-*tet*(X), DH5α-*tet*(X1), DH5α-*tet*(X2), DH5α-*tet*(X3) and DH5α-*tet*(X4) (Table 1). A total of 24 faeces and soil samples collected from chickens, pigs, and cattle farms from Sichuan Province were then used for further evaluation (Fig. 1).

### DNA extraction

Bacteria were incubated at 37 °C in Brain Heart Infusion broth with agitation at 200 rpm to achieve enough colonies for DNA extraction. Hipure Bacterial DNA Kit (Magen, Guangdong, China) were used to extract bacterial genome according to the manufacturer’s instruction. The DNeasy PowerSoil (Qiagen, Hilden, Germany) was used to extract metagenomic DNA from faeces and soil.

### Primer and probe design

The nucleotide sequences of *tet*(X3) and *tet*(X4) were obtained from GenBank. The specific real-time PCR primers and TaqMan probes for these genes (Tables 2 & 3) were designed using Primer Express software (ABI-Applied Biosystems Incorporated, Foster City, CA), and the NCBI Primer-BLAST module (https://www.ncbi.nlm.nih.gov/ tools/primer-blast/) was used to initially validate their specificity. Then, conventional and real-time PCR were both conducted to evaluate the specificity of primers and probes.

**Table 2:** Primers for real-time PCR detection of *tet*(X3) and *tet*(X4) genes

Primer	Sequence (5′-3′)	Gene	Product length	Accession No.	Reference
*tet*(X3)-qF	CAGGACAGAAACAGCGTTGC	*tet*(X3)	179 bp	MK134375.1	This study
*tet*(X3)-qR	GCAGCATCGCCAATCATTGT				
*tet*(X4)-qF	TTGGGACGAACGCTACAAAG	*tet*(X4)	181 bp	MK134376.1	This study
*tet*(X4)-qR	CATCAACCCGCTGTTTACGC

**Table 3:** Probes for detection of *tet*(X3) and *tet*(X4) genes

Probe	Sequence (5′-3′)	Gene	Accession No.	Reference
*tet*(X3)-P	AAGATTTTCCAAATGGAGTGAAG	*tet*(X3)	MK134375.1	This study
*tet*(X4)-P	TCGTGTGACATCATCT	*tet*(X4)	MK134376.1	This study

### Standard curves and PCR conditions

The *tet*(X3) and *tet*(X4) *g*enes were cloned into the pMD19-T vector separately, and then transferred into DH5α cells. Standard curves were established using real-time PCR on a QuantStudio™ 7 Flex Real-Time PCR System (ABI-Applied Biosystems Incorporated, Foster City, CA) using 10-fold serial dilutions of the recombinant plasmid DNA with original concentration of 47 ng/μL and 39.4 ng/μL for *tet*(X3) and *tet*(X4), respectively. Multiplex PCR reactions were performed in a total reaction volume of 20 μL, including 0.4 μL of each primer, 0.4 μL of probe, 0.4 μL of Passive Reference Dye (50×) (TransGen Biotech, Beijing, China), 10 μL of 2× TansStart® Probe qPCR SuperMix (TransGen Biotech, Beijing, China), 1.0 μL of DNA template, and 7.4 μL of ddH_2_O. Each pair of primers and probes were optimized to a final concentration of 0.2 pM. Real-time PCR reaction conditions were as follows: a cycle of 95 °C for 30 s, followed by 40 cycles of 95 °C for 5 s and 55 °C for 30 s.

### Specificity and sensitivity tests

To evaluate the specificity of the proposed method, DH5α strains containing *tet*(X), *tet*(X1), *tet*(X2), *tet*(X3) and *tet*(X4), respectively, were used to conduct the real-time PCR assay (Table 1). Genomic DNA extracted from bacteria, faeces and soil samples from different origin was then used to further validate the specificity and sensitivity of the method.

## Data Availability

The datasets supporting the conclusions of this article are included within the article. The data and materials used and/or analyzed during the current study are available upon reasonable request to the corresponding author.

## Abbreviations

PCR: Polymerase chain reaction
AMR: Antimicrobial resistance
XDR: Extensively drug resistant
WHO: World Health Organization
FDA: Food and Drug Administration
MIC: Minimal inhibitory concentration
*NCBI*: National Center for Biotechnology Information
*BLAST*: Basic Local Alignment Search Tool
CT: Cycle threshold
*16S rRNA*: 16S ribosomal Ribonucleic Acid
MDR: Multi-drug resistant
qPCR: Quantitative polymerase chain reaction
MALDI-TOF: Matrix-assisted laser desorption ionization-Time of Flight
